# Our Professional Future

**Published:** 1903-09-15

**Authors:** C. A. Van Duzee


### OUR PROFESSIONAL FUTURE.

BY C. A. VAN DUZEE, D.D.S.

All my life, as a professional man, I have noticed that one of the things we need in the dental profession is to get closer together. We need a larger supply of the milk of human kindness, and I think we want to develop the best that is in us. We have some progressive men who realize, individually, the great magnitude of the subject treated in this paper, but I presume this paper will probably not reach the men it should.

Every thinking man governs himself largely with reference to the future. Some carry the habit to an extreme, but many give it little consideration. In order that we may grasp those things which are essential to a proper understanding of the subject, we must examine ourselves carefully, our environment, those things which hinder us and those which may help us, and the fact must not be lost sight of that this profession of ours is not going to stand still and wait for us very long. There are influences at work that are beyond our control. The people at large are not standing still; the children of to-day are not the children of yesterday, and there is the handwriting on the wall for those of us who have eyes to see. Let us consider a few of the many conditions that surround the problem.

The question of ethics in dentistry has little, if anything, to do with the "Code of Ethics" as promulgated for many years and this same "Code of Ethics" has, in my opinion, been one of the greatest stumbling-blocks in the way of the establishment of a true ethical atmosphere in our professional circle.

When a youth receives his education, whether it be obtained in the best colleges, in the business world, or among the cattle upon the farm, the result is apt to be a creature of circumstances and environment, with a conception of right and wrong, more or less colored by the associations of the daily life of the individual. The influences of the parents, the school fellows and the business acquaintances, together with the hereditory temperament and numbers of other things, all combine to produce a man of a certain worth or value from a social standpoint. One inherits a combination of vicious traits of character, another refinement and virtue of a high order; the first, through an inherent tendency to progress overcomes in some degree the handicap of birth; the second, through weakness of character, descends to a level with his surroundings. The world is composed of an endless variety of men who range from one extreme to the other. The one who rises above his normal level, even if he does not attain to the ultimate plane of the one who falls below his natural position, deserves more or less credit for his accomplishments; and again, the one who falls below deserves more or less censure.

The people at large are the judge, self-constituted, perhaps, before whom each man must stand. In a consideration of this question from a dental point of view, one is apt to forget all these things, and to weigh and classify men by comparison according to a certain standard· Our friend across the way never graduated from a reputable school; his normal condition is bad; he does not do good work, at least we have seen some that was not up to the mark. His fees are below the line, and he does not grace the profession to which he has obtained admission. Let us go back along his trail In youth he was the mainstay of his parents in an humble walk of life, and until many of tl e best years of his life had passed, the spark of ambition which glowed within his soul was all but smothered; at last the chance comes to him to knock at the door of his chosen calling. The knock is feeble, perhaps from lack of many things which to others were not denied. He enters and assumes a place. Go into his home life to-day; perhaps you will find a wife who came with him out of the past. There may be children born of this union and dependent upon this man; you may find them studious and affectionate, and yet they are not well dressed or polished, but as compared with those other children who lived in the past, they are positive evidence of progress in every way. Who shall say that this man is not entitled to be received upon an equal footing with him, who, while adding grace to the professional calling which has become his through the very fact that by birth, environment, and fortunate circumstances, is the better fitted to demand admission, even though the present status of this second man is below that to which, by very right, he was originally entitled?

We are apt to judge our fellows superficially. We see them from a certain point of observation, and above all, we see them with eyes that are our own.

A man makes an appointment with a child lacking in everything that goes to make a good patient, and puts his very life into the operation and the attempt to educate that child for future operations. The results are not beautiful to look upon, nor are they of much value to the tooth. The operator leaves the chair sick at heart, discouraged, and physically worn. You, my friends, may see this case next fall. As you look upon this faulty piece of work, and may be the only one you will ever see from the man across the way, you will be able to put yourself exactly at the right point of observation to see this picture as a whole, or will you see the failure through glasses that are colored? Will you say to the child or its mother, and perhaps her friends, that the work is a failure and must all be done over? Will you take this child, now older and perhaps improved as a patient by the lessons so patiently taught by the man across the way, and perhaps in many ways better able to meet you half way to the end that a better operation may result? Will you steal the life-blood of your neighbor and use it without credit to him? Will you classify him by the result of this operation, by the clothes he wears, or the style of sign he displays upon his office window? A dentist once placed a cotton dressing in a tooth and sealed it with cement under circumstances which demanded exactly that treatment, and was called away from the city. The man who was called upon to finish that case, and it had been neglected for months, removed the cement, found the cotton in the pulp canal, and before several people who happened to be in his office, criticised his brother severely. Another dentist received a patient from another city, and observing a crown which had been worn through by a sharp occluding cusp, although only a year old, remarked to his patient that he must reset that crown. He took it off, made a new one, and placed it in position without charge and without comment to the patient, and when pressed by another for an explanation, said: "Dr. So and So is a good man and deserves a good turn." I know these men well; the first a graduate of the best schools, of good family, and having every opportunity for the development of the most that he was capable of; the other man, of no family, of humble birth, and the only education he ever had was picked up as best it could be obtained, and he practices dentistry to-day without a degree.

I have been much interested in an article by Dr. F. R. Adams, of Vernon, N. Y., upon Dental Ethics, published in the *Cosmos*, for August, 1901, and I venture to quote the last paragraph of this delightful essay.

The doctor says: "Thus endeth the Code of Ethics. Now what does it all amount to? The greatest ethical law ever written, the one which includes all the teachings of the code, and in fact, of all ethical law, is 'Do unto others as you would that others should do unto you.' If we will only keep this maxim before us and try to live up to the best that is in us, we will not need any Code of Ethics. 'Honesty is the best policy,' and while the dishonest man may seem to flourish to-day, yet the man who conducts his practice in a straightforward, honest manner, will prosper, and he will have, besides, that greatest of all blessings, a clean conscience. This above all, 'To thine own self be true, and it must follow as the night the day, thou canst not then be false to any man.' "

The next line of the page informs us that there was no discussion of this paper. Men write upon everything and anything, and there are many to discuss the subjects, but when a man has the courage to write upon a subject that has more to do with the future of the dental profession than any other thing, there is no discussion.

I want to say a word on the question of advertising. What have we to advertise? Do we wish to attract to our places of business people who want something for nothing? Do we wish to attempt to create in the minds of our prospective patients the idea that we are more skillful than our neighbor? Do we wish to place our services at their disposal for fees which of themselves will attract; or do we wish to develop a legitimate circle of patrons? It lies with us, and in exactly the manner of our greeting to the public will our future life develop. There is but one legitimate method of advertising, and that is, that we are dentists; and our office is in a certain location, and that we are there for practice between certain hours.

Some one must care for the poor, the ignorant, and the selfish, and it is the duty of every professional man to give freely of his time to the poor, and he must regulate his fees up and down the line to meet the requirements of his practice. That does not mean that he should rest there.

The strugling young person of to-day, if treated honestly, may be educated into the most desirable kind of a patient for the future, and one of the greatest pleasures of our lives is in contemplating the results of our work as years go by, and the youth of yesterday becomes the sinew of to-day's business and social world, provided the operations were well done and the youth well educated along the lines that fall to us as members of a young and little known profession.

There is another question that is very closely related to my subject: What shall we do to counteract the influences of our friends who are neither honest nor ethical? There are three ways in which we may legitimately attack the men who are without the pale of professional life in dentistry.

We may set them an example which will comand respect. We may stop throwing mud at them, and do a little true missionary work by inviting them to be one of us, and we may provide clinics in which the poor, the ignorant, and the selfish may be properly treated, at prices within their means. What large city has not its free dispensary and its hospital for those who are unfortunate? What large city has an infirmary or hospital for dental work, unless there be a department of some college of dentistry to take its place? We owe it to our profession to see that these things are accomplished, and yet how little is being done. We have not the time, the brains, the business ability, or the courage to go out and do what every dentist knows would do more to further the best interests of dentistry and humanity than tons of paper and barrels of ink will ever do.

Our brothers in medicine and law know these things, and are doing much to organize and support such enterprises. How long are we going to trust to luck to help us to this work that is crying out for help? In this city we have physicians and surgeons and hospitals, supported in whole or in part by the government, to care for the unfortunate. How many quacks will such things deprive of their victims? There are men who are paid to spend their whole time in the courts and the highways in looking after the criminal or unfortunate classes. How many shyster lawyers are deprived of their victims through these methods? If there was a public infirmary in this city for charitable or semi-charitable patients who require dental services, many quack dentists would be deprived of the excuse for their existence.

There is another duty which demands more consideration. Dentistry encroaches and follows closely upon the practice medicine, and the physician and surgeon are worthy of cultivation. To the ethical man they are ever willing to extend courtesy and brotherly advice. We need a closer relationship, and the accomplishment of that requires two things. First, we must know our own professional business well enough to command their respect; and, secondly, we must go to them in consultation. The science of medicine and surgery is old enough to make it our duty to take the initiative, and the results of a more intimate acquaintance can not but further the best interests of dentistry, and in addition the benefits to the public must of necessity be very large.

These things all bear a close relationship to the subject of this paper. The ethical man in any profession lives in an ethical atmosphere, the possibilities for which must depend largely upon the standing of the whole. A few men of worth, character, and ability, cannot elevate the whole profession to that point where it should be to-day, and as time passes, the future will demand more of us. The field is broadening, the requirements are becoming more exacting, the public is becoming educated, and upon every hand the dentist is brought face to face with more difficult problems year after year.—*Review.*

*Comment by the editor of the Dental Review.*—It is so easy to censure when the facts in the case are not all at hand, and they are seldom at hand when the second dentist sees the work. I have reached the point where I positively will not take a fellow dentist to task no matter what character of work I see from his hands unless there is unmistakable evidence of gross and palpable neglect amounting to malpractice, and even then I take him to task with reservation and never with the knowledge of the patient.

There are some individuals for whom to do good work at particular times is impossible. And the deceptive thing about it is that frequently for these same patients at another time good work is easy of accomplishment. We have all seen this, and why is it that we will not render to our predecessor the same meed of charity that we would ask for ourselves under similar circumstances? The bedevilment of professional comity and ethics relates not so much to violations of the written code of ethics as it does to underhanded insinuations made against fellow practitioners before patients. We should of course at all times defend a patient against the malpractice of an unscrupulous dentist, but we can do this without comment or criticism. Our duty to a patient is simply to remedy any wrong which may have been done as best we can and not discuss the author of the wrong.

Above all things, let us cultivate charity for the shortcomings of others, especially when we have no means of knowing that apparent lapses are not accidental or unavoidable instead of deliberate and censurable. It is the basest kind of professional perfidy to always seek to undermine the reputation of a fellow practitioner, and the irony of fate usually wills in that the undermining reacts on the one who attempts it rather than the one against whom it is aimed.

